# Cleavage of delta and epsilon tubulin PCR products is observed upon PCR purification

**DOI:** 10.17912/micropub.biology.000980

**Published:** 2023-10-10

**Authors:** Prasad Parab, Jyoti Iyer, Tyler W Johannes

**Affiliations:** 1 Chemical Engineering , University of Tulsa, Tulsa, Oklahoma, United States; 2 Chemistry and Biochemistry , University of Tulsa, Tulsa, Oklahoma, United States; 3 Chemical Engineering, University of Tulsa, Tulsa, Oklahoma, United States

## Abstract

In this study, we report an unusual phenomenon of the self-cleavage of purified PCR products of codon-optimized *Chlamydomonas reinhardtii* delta tubulin ( *uni3* ) and epsilon tubulin ( *bld2* ) genes through an unknown mechanism. Our studies revealed that intact PCR products for both these genes could be obtained upon PCR amplification from plasmid templates carrying these genes. However, interestingly, purification of these PCR products led to their cleavage through an unidentified mechanism. This cleavage persisted despite using different PCR purification kits. Deleting a synthetic intron within the delta tubulin gene also did not have any effect on this cleavage.

**Figure 1.**
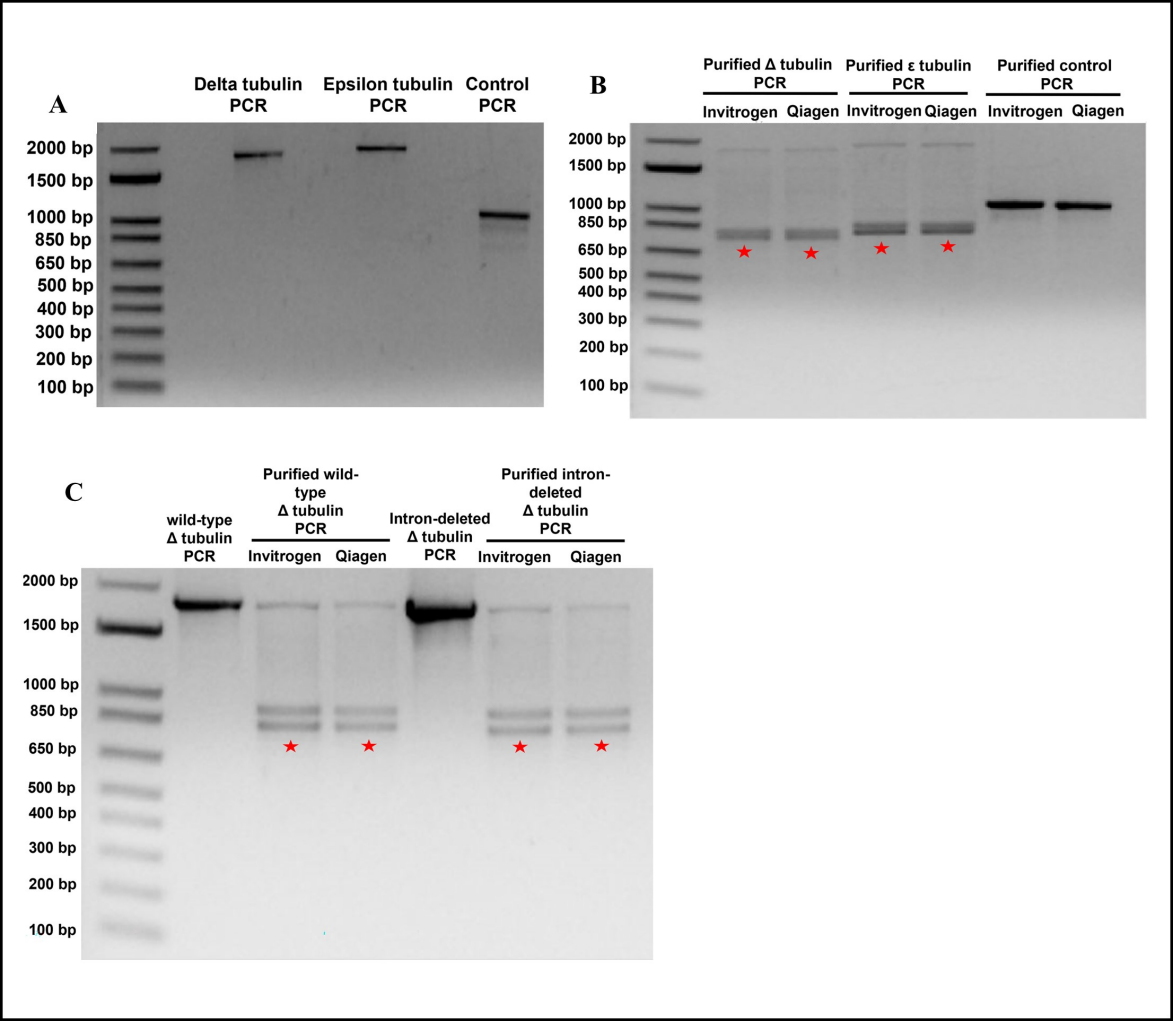
Full-length PCR products of codon-optimized *Chlamydomonas reinhardtii* delta and epsilon tubulin genes were successfully obtained upon PCR. However, PCR purification of these PCR products led to their cleavage through an unknown mechanism. A) Agarose gel electrophoresis of delta tubulin ( *uni3* ), epsilon tubulin ( *bld2* ) and positive control ( *dyn-1* ) PCR products showing that PCR products of the expected sizes of 1775 bp for delta tubulin ( *uni3* ), 1874 bp for epsilon tubulin ( *bld2* ) and 1011 bp for the positive control ( *dyn-1* ) were obtained. B) Purification of the delta and epsilon tubulin PCR products using either the Invitrogen Purelink™ Quick PCR purification kit (Thermo Fischer Scientific, Waltham, MA) or the Qiagen Qiaquick® PCR purification kit (Qiagen, Redwood City, CA) led to cleavage of the PCR products as seen by the appearance of lower sized bands between 650 bp and 1000 bp upon agarose gel electrophoresis (red stars). Importantly, the PCR product of the positive control ( *dyn-1* ) behaved as expected and did not exhibit cleavage upon purification with either kit. C) Agarose gel electrophoresis showing that the deletion of a synthetic intron close to the identified cleavage junction within the sequence of delta tubulin did not eliminate cleavage of the delta tubulin PCR product upon PCR purification with either kit (red stars).

## Description

Centrosomes are the major microtubule organizing centers of most animal cells. They contribute to a variety of cellular functions including spindle assembly, cell polarity, cell shape and cilia assembly (Reviewed in Nigg *et al.* , 2009; Bornens *et al.,* 2012; Conduit *et al.,* 2015; Fu *et al.,* 2015; Nigg and Holland, 2018). Centrosomes consist of orthogonally-paired cylindrical centrioles enveloped by the protein-dense pericentriolar material (Reviewed in Schwarz *et al.,* 2018). *Chlamydomonas reinhardtii* delta and epsilon tubulin proteins that are coded by the *uni3* and *bld2* genes respectively, play a crucial role in maintaining the proper triplet microtubule architecture of centrioles (Dutcher and Trabuco, 1998; Dutcher *et al.,* 2002). To obtain further insights into the functions of these genes in regulating centriole architecture, we wanted to generate purified PCR products of *Chlamydomonas reinhardtii* delta and epsilon tubulin cDNAs carrying synthetic introns that were codon-optimized for expression in *C. elegans* . These purified PCR products would enable us to perform downstream functional assays to further characterize the functions of these genes. Although we were successful in obtaining full length PCR products of codon-optimized *Chlamydomonas reinhardtii* delta and epsilon tubulin genes **( Figure 1A )** , these PCR products exhibited an unexpected cleavage upon their purification using either the Invitrogen Purelink™ Quick PCR purification kit or the Qiaquick® PCR purification kit **( Figure 1B ).** As shown in **Figure 1B** , both the delta and epsilon tubulin PCR products but not a non-relevant control PCR product ( *dyn-1* ) exhibited cleavage after their purification using the Invitrogen Purelink™ Quick PCR purification kit or the Qiaquick® PCR purification kit. Purification of the delta and epsilon tubulin PCR products using a non-kit-based isopropanol precipitation method as well as upon their gel extraction using a Qiaquick Gel Extraction kit (Qiagen, Redwood City, CA), both yielded this similar pattern of cleavage. To confirm that the cleaved fragments indeed correspond to the PCR products of the delta and epsilon tubulin genes, DNA sequencing of these lower sized fragments was performed after excising these separated bands from the agarose gel. Our sequencing results confirmed that the lower sized fragments that were observed between 650 bp and 1000 bp upon agarose gel electrophoresis of the purified delta and epsilon tubulin PCR products **( Figure 1B )** contained DNA sequences from these respective genes. Our sequencing analysis identified a synthetic intron close to the genomic region where the delta and epsilon tubulin PCR products were being cleaved. Since the sequence of this synthetic intron was identical between the delta and epsilon tubulin genes, we questioned whether the presence of this synthetic intron was responsible for the cleavage of the purified PCR products of these genes. To address this, site-directed mutagenesis was performed to remove this synthetic intron from the sequence of the delta tubulin gene and the PCR purification procedure was repeated. As shown in **Figure 1C,** deletion of the synthetic intron close to the cleavage region within the delta tubulin gene did not eliminate cleavage of the purified PCR product. These data indicate that this synthetic intron does not contribute to this observed cleavage phenomenon. In the future, studies should be directed to further characterize the molecular mechanisms mediating the cleavage of the *Chlamydomonas reinhardtii* delta and epsilon tubulin PCR products upon purification. Since we have invested a considerable amount of time and resources on this project, we would like to share these findings with the scientific community and with other researchers working on these genes to prevent a further loss of their valuable time and resources.

## Methods

I) PCR conditions, analysis, and purification

PCR was performed using a C1000™ Thermal Cycler (BioRad, Hercules, CA). 35 cycles of amplification were used for 50 µL PCR reaction volume consisting of:

1 µL- 10 µM forward primer, 1 µL- 10 µM reverse primer, 35 ng- plasmid template ( *uni3* , *bld2* , *dyn-1* ), 1 µL - 10 mM dNTPs, 2 µL- 50 mM MgSO _4_ , 5 µL - 10X Hi-Fi Buffer, 0.2 µL - Platinum® *Taq* DNA Polymerase Hi -Fi enzyme, and molecular biology water was used to bring the final volume to 50 µL.

PCR conditions for *uni3* / *bld2* :

Initial denaturation: 94°C - 60 s

Denaturation: 94°C - 15 s

Annealing: 55°C - 30 s

Extension: 68°C - 60 s

Final Extension: 72°C - 5 min

The control PCR product ( *dyn-1)* was obtained from a synthesized plasmid construct that contains the coding sequence for blue fluorescent protein with homology arms to the *C. elegans dyn-1* gene.

PCR conditions for the control ( *dyn-1* ) PCR:

Initial denaturation: 94°C - 60 s

Denaturation: 94°C - 15 s

Annealing: 53°C - 15 s

Extension: 72°C - 75 s

Final Extension: 72°C - 5 min

To verify the results of PCR amplification, the PCR products were analyzed by gel electrophoresis on a 1% agarose gel. The PCR-amplified products were further purified using Invitrogen Purelink™ Quick PCR purification kit and Qiaquick® PCR purification kit. Note that molecular biology grade water was used to elute the PCR products in the final step for PCR purification using both these kits instead of the elution buffer. Upon elution, the purified PCR products were analyzed by gel electrophoresis on a 1% agarose gel.

II) Site-directed mutagenesis

For performing the site-directed mutagenesis, the PCR reaction mixture comprised of all the same components used in PCR amplification of the *uni3* gene except that the Phusion® High-Fidelity DNA Polymerase was used for PCR amplification.

PCR conditions for delta tubulin intron deletion using site-directed mutagenesis:

Initial denaturation: 98°C - 30 s

Denaturation: 98°C - 10 s

Annealing: 68.6°C - 30 s

Extension: 72°C - 6 min

Final Extension: 72°C - 10 min

Site-directed mutagenesis was followed by digestion with 5 units of Dpn1 for 2 hours, followed by bacterial transformation and plasmid purification using the QIAprep® Spin Miniprep kit. The isolated plasmid samples were sent for whole plasmid sequencing (Plasmidsaurus LLC, Eugene, OR). Upon analyzing the sequencing results, the plasmid with successful intron deletion was selected and used for further analysis.

## Reagents

**Table:** 

Reagents	*Supplier*	Catalog number
1. *Chlamydomonas reinhardtii* Delta tubulin (1775 bp; *uni3* gene) and Epsilon tubulin (1874 bp; *bld2* gene) cDNAs that were codon optimized for expression in *C. elegans* were synthesized in the cloning vector pUC-GW-Kan	Genewiz Inc. (Burlington, MA)	-
2. The positive control ( *dyn-1* ) plasmid was synthesized in the cloning vector pUC-GW-Amp	Genewiz Inc. (Burlington, MA)	-
3. Forward and Reverse primers	Integrated DNA Technologies, Inc.(Coralville, IA)	-
4. 10 mM dNTP Mix	Thermo Fisher Scientific™ (Waltham, MA)	R0192
5. 50 mM MgSO _4_	Thermo Fisher Scientific™ (Waltham, MA)	Included in 11304011
6. 10X Hi-Fi Buffer	Thermo Fisher Scientific™ (Waltham, MA)	Included in 11304011
7. Platinum® *Taq* DNA Polymerase Hi -Fi enzyme	Thermo Fisher Scientific™ (Waltham, MA)	11304011
8. Molecular biology water	-	-
9. Dpn1 enzyme	Thermo Fisher Scientific™ (Waltham, MA)	ER1701
10. Phusion® High-Fidelity DNA Polymerase	New England BioLabs *Inc.* (Ipswich, MA)	M0530S
11. Invitrogen™ 1 Kb Plus DNA Ladder	Thermo Fisher Scientific™ (Waltham, MA)	10787026
12. Luria-Bertani (LB) media	· 600mL ddH _2_ 0 · 15 g Difco™ LB Broth, Lennox (BD, Franklin lakes, NJ) · Autoclave at 121°C for 15 mins	240220
13. 10 mg/mL Kanamycin solution	IBI Scientific (Dubuque, IA)	IB02120
14. Invitrogen Purelink™ Quick PCR purification kit	Thermo Fischer Scientific™ (Waltham, MA)	K310001
15. Qiaquick® PCR purification kit	Qiagen (Redwood City, CA)	28104
16. QIAprep® Spin Miniprep kit	Qiagen (Redwood City, CA)	27104
17. Agarose I™	VWR® International (Radnor, PA)	97062-244
18. QIAquick Gel Extraction Kit	Qiagen (Redwood City, CA)	28704

Primers and sequences used for study

I) Primers for PCR amplification of Epsilon tubulin

Forward primer: 5’- ATT CGA ATA TAT ATT GTC AGT TG -3’

Reverse primer: 5’- ATA CGA GGA TTA TGG TAC AAG -3’

II) Primers for PCR amplification of Delta tubulin

Forward primer: 5’- TTG TTT CTT TCT TTT AAT GTT AAA TAT TTC CAG AAC TAT GCC ATG -3’

Reverse primer: 5’- GAT AAA ATA ATT ATT CGG GCA GTA ATA AAA CAG GGA TCT ATC ACT TC -3’

III) Primers for site-directed mutagenesis for Delta tubulin with one intron deleted

Forward primer: 5’- TCG AGG AGG CCG GAC TCA AGG GAC AAT CCT CCG GAC CAG G -3’

Reverse primer: 5’- CCT GGT CCG GAG GAT TGT CCC TTG AGT CCG GCC TCC TCG A -3’

IV) Primers for amplification of control *(dyn-1)* :

Forward primer: 5’- AAA ATC GAT TTT CAG GTA GTT CAG C -3’

Reverse primer: 5’- TTG ATC ACA GGG ATC AAC GCC -3’

V) Sequence of *Chlamydomonas reinhardtii* delta tubulin ( *uni3* ) cDNA codon optimized for expression in *C. elegans* (contains synthetic introns (lowercase + underlined) and homology arms for CRISPR genome editing (only lowercase)):

5’-ttgtttctttcttttaatgttaaatatttccagaactATGCCATGCATCACCCTCCAACTCGGACAATGCGGAAACCAACTCGGATGCTCCCTCTTCAACACCCTCGCCACCGAGTTCTCCTCCCACGACTACGGAACCGACGCCGTCCACGAGTACTTCCGTCCATCCGCCGACCCAAACCTCTACACCGCCCGTTCCGTCCTCATCGACATGGAGCCAAAG gtaagtttaaacatatatatactaactaaccctgattatttaaattttcag GTCGTCGCCGGAGCCCGTTCCGCCGCCGCCGCCTCCGGATCCTGGTGGCGTTACCCATCCTCCGGATACCTCGTCATGCAATCCGGATCCGGAAACAACTGGGCCCAAGGATTCCACGGATACGGACCACAAGTCCACGAGGACGCCCTCGACCTCGTCCGTAAG gtaagtttaaacagttcggtactaactaaccatacatatttaaattttcag GAGGTCGAGCACGCCGACTCCCTCACCGGATTCCTCCTCCTCCAATCCATGGCCGGAGGAACCGGAGCCGGACTCGGAACCTACGTCGCCGAGGCCCTCCGTGACGAGTACCACTCCGCCTTCGTCGCCAACTGCTGCGTCTGGCCATACGAGTCCGGAGAGGTCATCGTCCAACCATACAACACCCTCCTCACCCTCTCCCACCTCGCCGACGTCTCCGACGGACTCGTCCTCCTCGAGAACGAGGCCCTCCACCGTACCGCCGCCAAGCTCTACGGAATCGCCCGTCCATCCTTCGGAGTCCGTGGACGTGTCCTCGGACGTGCCGGAGAGTCCCGTGTCGAGGAGGCCGGACTCAAG gtaagtttaaacatgattttactaactaactaatctgatttaaattttcag GGACAATCCTCCGGACCAGGAGGATGGGGAGTCTGCACCGCCCCACTCGCCGAGCTCGTCACCCGTCTCTGCGGACACCCAGCCTACCGTCTCCTCACCCTCCGTTCCGTCCCACAACTCCCACCAGCCAACATCGACTTCACCACCTTCACCTGGCCAGCCCTCACCAAGCGTCTCCGTCAAATGCTCGTCACCGGATCCGTCCTCGAGGAGGGACTCGACTGGTCCATCACCCCACAATCCCCAGGAGCCGCCGCCGCCCTCGGAGCCGGACTCGCCGGACCAACCGTCAACCGTGCCCTCGCCTCCTGGCTCATCCTCCGTGGACAAGGAGCCGCCGAGGCCGACGTCGGAGAGTTCGCCGACCCAGCCCTCTCCGCCGCCTGGTCCCCAGAGCCACTCTCCGTCTCCTACTCCACCGGACGTTTCGGACGTTGCGCCATGTCCGCCTGCCTCCTCTCCAACGACCGTCACTGCGTCGGACCAATCCAACGTATGCAAGAGCACGCCTACGGAATGCTCGAGTCCCGTGCCTTCGTCCACCAATACGAGAAGTACGGACTCTCCGTCGCCGAGTTCCAAGACTGCTTCGCCCGTATCGAGGACATCGCCCAACGTTACGCCCGTCTCGACTACAAAGACCATGACGGTGATTATAAAGATCATGATATCGATTACAAGGATGACGATGACAAGATGCCTAAAGATCCAGCCAAACCTCCGGCCAAGGCACAAGTTGTGGGATGGCCACCGGTGAGATCATACCGGAAGAACGTGATGGTTTCCTGCCAAAAATCAAGCGGTGGCCCGGAGGCGGCGGCGTTCGTGAAGtgatagatccctgttttattactgcccgaataattattttatc-3’

VI) Sequence of *Chlamydomonas reinhardtii* epsilon tubulin ( *bld2* ) cDNA codon optimized for expression in *C. elegans* (contains introns (lowercase + underlined) and homology arms for CRISPR genome editing (only lower case)):

5’- attcgaatatatattgtcagttgttctgtttgtcgtcgtgATGCCACGTGAGCTCGTCACCATCCAAGTCGGACAATGCGGAAACCAAGTCGGATGCCGTTTCTGGGAGCTCGCCCTCCGTGAGCACGCCGCCTACAACACCAAGGGAGTCTACGACGAGGCCCTCTCCTCCTTCTTCCGTAACGTCGACACCCGTGTCGAGCCACCACGTAACCTCCCAGTCGGAGAGGGACGTGGAGCCATCCGTACCCTCAAGGCCCGTTCCGTCATCGTCGACATGGAGTGCGGAGTCATCAACGAGATGCTCAAGGGACCACTCGGAGAGGTCCTCGACACCCGTCAACTCGTCTCCGACGTCTCCGGAGCCGGAAACAACTGGGCCCACGGACACCACGAGTACGGACCACGTTACCACGACGCCATCCTCGACAAG gtaagtttaaacatatatatactaactaaccctgattatttaaattttcag ATCCGTCTCACCGCCGAGGACTGCGACTCCCTCCAATCCTTCATGGTCCTCCACTCCCTCGGAGGAGGAACCGGATCCGGAGTCGGAACCTACATCGTCCGTATGCTCGCCGACGAGTTCCCAGGAGTCTTCCGTTTCACCGGATCCGTCTTCCCATCCGAGGACGACGACGTCGTCACCTCCCCATACAACGCCATGCTCGCCCTCGGACAACTCGTCGAGCACGCCGACTGCGTCCTCCCAATCGAGAACCAAGCCCTCATCGACATCGTCAACCGTACCGAGGCCGCCCGTGACCGTGCCGCCGCCGCCGACGCCGCCGCCTCCGCCGTCTCCGGACTCAAG gtaagtttaaacagttcggtactaactaaccatacatatttaaattttcag GGATCCGGAGGAGGATCCAAGCCATTCGACTCCATGAACGGAGTCGCCGCCTCCCTCCTCCTCCACCTCACCGCCTCCGTCCGTTTCGAGGGACCACTCAACGTCGACCTCAACGACATCACCATGAACCTCGTCCCATACCCACGTATGCACTTCCTCCTCTCCTCCATGTCCCCACTCCAACCACCACCAAAGGACAAGGACCCACGTACCCTCGACCAAGTCCGTGTCTTCGGAGACGTCTTCTCCCGTGAGCACCAACTCATCCGTGCCGACCCACGTGCCGCCACCTACCTCGCCTGCGGACTCATCGCCCGTGGACCAACCGCCACCATGGCCGACATCAACCGTAACGTCGCCCGTCTCCGTCCACAACTCAAG gtaagtttaaacatgattttactaactaactaatctgatttaaattttcag ATGGTCCACTGGAACTCCGAGGGATTCAAGCTCGGAATCTGCTCCACCCCACCAGTCGGATGCCCATTCGGACTCCTCTGCCTCGCCAACAACACCGCCATCGCCCACACCTTCACCACCATGCGTGAGCGTTTCGACAAGCTCTACAAGCGTCGTTTCTACACCCACCACTACGAGCAATACATGGACCCAGGAGGATTCACCTCCGCCATGGAGGTCGTCGGAGACCTCACCGCCCAATACCGTGCCCTCGAGGGAGCCACCCAAGCCCCACCACTCACCCGTCTCCGTCCACGTGGACTCTCCTTCCTCCCAGGAGGTTCCGGTGGTTCTGGTGGATCCGGTAAGCCTATCCCAAATCCTTTGTTGGGTCTGGACTCCACGATGCCTAAAGATCCAGCCAAACCTCCGGCCAAGGCACAAGTTGTGGGATGGCCACCGGTGAGATCATACCGGAAGAACGTGATGGTTTCCTGCCAAAAATCAAGCGGTGGCCCGGAGGCGGCGGCGTTCGTGAAGtgataatattcatttaatccaacttgtaccataatcctcgtat-3’

VI) Sequence of the positive control used in this study *(dyn-1)* :

5’- AAAATCGATTTTCAGGTAGTTCAGCGTATAACCACCAGGATCAGCGATGGTTTCGGAATTGATTAAAGAAAATATGCACATGAAGCTCTACATGGAGGGAACCGTCGACAACCACCACTTTAAATGTACCTCCGAGGGAGAGGGAAAGCCATACGAGGGAACCCAAACCATGCGTATCAAGGTCGTCGAGGGTGGTCCGCTCCCATTCGCCTTTGATATCCTCGCCACCTCCTTCCTCTATGGTTCCAAGGTAAGTTTAAACATATATATACTAACTAACCCTGATTATTTAAATTTTCAGACCTTCATCAACCACACCCAAGGAATCCCAGACTTTTTTAAACAATCCTTCCCAGAGGGATTCACCTGGGAGCGTGTCACCACCTACGAGGACGGAGGAGTCCTCACCGCCACCCAAGACACCTCCCTCCAAGACGGATGCCTCATCTACAACGTCAAGGTAAGTTTAAACAGTTCGGTACTAACTAACCATACATATTTAAATTTTCAGATCCGTGGAGTCAACTTCACCTCCAACGGACCAGTCATGCAAAAGAAGACCCTCGGATGGGAGGCCTTCACCGAGACCCTCTACCCAGCCGACGGAGGACTCGAGGGACGTAACGACATGGCCCTCAAGCTCGTCGGAGGATCCCACCTCATCGCCAACGCCAAGGTAAGTTTAAACATGATTTTACTAACTAACTAATCTGATTTAAATTTTCAGACCACCTACCGTTCCAAGAAGCCAGCCAAGAACCTCAAGATGCCAGGAGTCTACTACGTCGACTACCGTCTCGAGCGTATCAAGGAGGCCAACAACGAGACCTACGTCGAGCAACACGAGGTCGCCGTCGCCCGTTACTGCGACCTCCCATCCAAGCTCGGACACAAGCTCAACTACCCATATGATGTTCCAGATTACGCTGGAGGATCTGGAGGCGGTTCTGGCGGAGGTTCTGGTATGTCGTGGCAAAACCAGGGAATGCAGGCGTTGATCCCTGTGATCAA – 3’
